# [Cr^III^_8_Ni^II^_6_]^n+^ Heterometallic Coordination Cubes

**DOI:** 10.3390/molecules26030757

**Published:** 2021-02-02

**Authors:** Helen M. O’Connor, Sergio Sanz, Aaron J. Scott, Mateusz B. Pitak, Wim T. Klooster, Simon J. Coles, Nicholas F. Chilton, Eric J. L. McInnes, Paul J. Lusby, Høgni Weihe, Stergios Piligkos, Euan K. Brechin

**Affiliations:** 1 EaStCHEM School of Chemistry, The University of Edinburgh, David Brewster Road, Edinburgh EH3 5JF, UK; oconnoh7@tcd.ie (H.M.O.); s.calvo@fz-juelich.de (S.S.); Aaron.Scott@ed.ac.uk (A.J.S.); Paul.Lusby@ed.ac.uk (P.J.L.); 2 UK National Crystallography Service, Chemistry, Highfield Campus, University of Southampton, Southampton SO17 1BJ, UK; Mateusz.Pitak@matthey.com (M.B.P.); W.T.Klooster@soton.ac.uk (W.T.K.); S.J.Coles@soton.ac.uk (S.J.C.); 3 Department of Chemistry, The University of Manchester, Oxford Road, Manchester M13 9PL, UK; nicholas.chilton@manchester.ac.uk (N.F.C.); eric.mcinnes@manchester.ac.uk (E.J.L.M.); 4 Department of Chemistry, University of Copenhagen, Universitetsparken 5, DK-2100 Copenhagen, Denmark; weihe@chem.ku.dk

**Keywords:** molecular magnetism, supramolecular chemistry, heterometallic clusters, magnetometry, EPR spectroscopy

## Abstract

Three new heterometallic [Cr^III^_8_Ni^II^_6_] coordination cubes of formulae [Cr^III^_8_Ni^II^_6_L_24_(H_2_O)_12_](NO_3_)_12_ (**1**), [Cr^III^_8_Ni^II^_6_L_24_(MeCN)_7_(H_2_O)_5_](ClO_4_)_12_ (**2**), and [Cr^III^_8_Ni^II^_6_L_24_Cl_12_] (**3**) (where HL = 1-(4-pyridyl)butane-1,3-dione), were synthesised using the paramagnetic metalloligand [Cr^III^L_3_] and the corresponding Ni^II^ salt. The magnetic skeleton of each capsule describes a face-centred cube in which the eight Cr^III^ and six Ni^II^ ions occupy the eight vertices and six faces of the structure, respectively. Direct current magnetic susceptibility measurements on (**1**) reveal weak ferromagnetic interactions between the Cr^III^ and Ni^II^ ions, with *J*_Cr-Ni_ = + 0.045 cm^−1^. EPR spectra are consistent with weak exchange, being dominated by the zero-field splitting of the Cr^III^ ions. Excluding wheel-like structures, examples of large heterometallic clusters containing both Cr^III^ and Ni^II^ ions are rather rare, and we demonstrate that the use of metalloligands with predictable bonding modes allows for a modular approach to building families of related polymetallic complexes. Compounds (**1**)–(**3**) join the previously published, structurally related family of [M^III^_8_M^II^_6_] cubes, where M^III^ = Cr, Fe and M^II^ = Cu, Co, Mn, Pd.

## 1. Introduction

**Figure 1:**
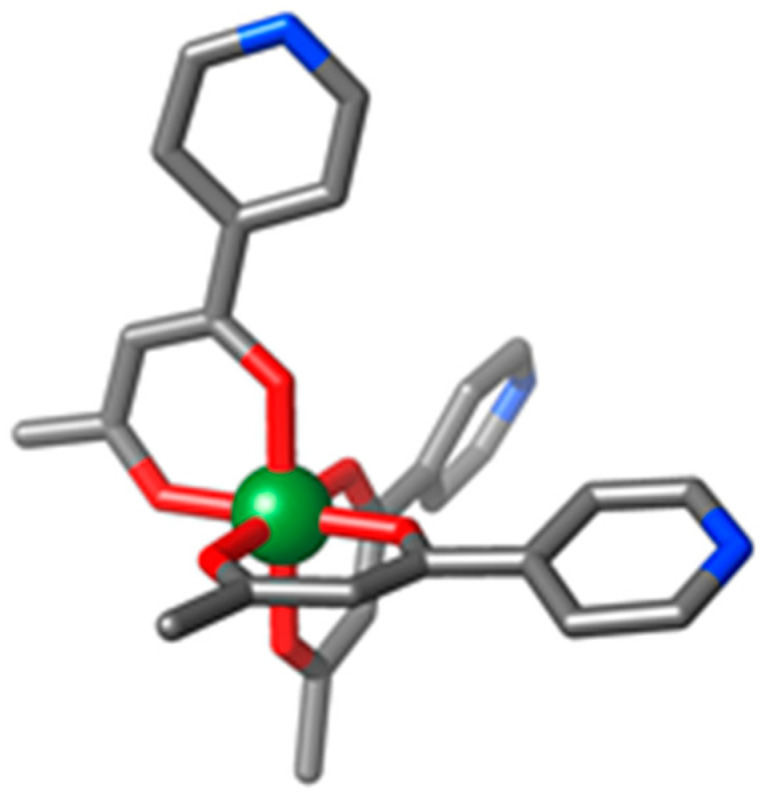
Molecular structure of [M^III^L_3_], where HL = 1-(4-pyridyl)butane-1,3-dione. Colour code: M^III^ = green, O = red, N = blue, C = grey. H-atoms have been omitted for clarity.

Heterometallic coordination complexes have seen application in areas as diverse as metalloprotein chemistry [1,2], catalysis [3], porous materials [4,5], and magnetism [6]. The latter includes three-dimensional (3D) networks [7], two-dimensional (2D) sheets [8], one-dimensional (1D) chains [9], and zero-dimensional (0D) (molecular) polygons and polyhedra [10,11], investigating controllable exchange interactions [12], enhanced magnetocaloric effects [13], spin frustration [14], slow relaxation of the magnetisation [15,16], and quantum coherence timescales [17]. A search of the Cambridge Structural Database (CSD) reveals that heterometallic wheels of varying size and metal ratios dominate the chemistry of polymetallic clusters containing both Cr^III^ and Ni^II^ ions with a nuclearity of four or more. Examples include [Cr_7_Ni], [Cr_9_Ni], [Cr_8_Ni_2_], [Cr_7_Ni_2_], [Cr_6_Ni_2_], [Cr_2_Ni_5_], [CrNi_6_] wheels and discs (centred/Anderson wheels), and [Cr_14_Ni_2_] and [Cr_28_Ni_4_] ‘linked rings’ [18,19,20,21,22,23]. Surprisingly, the search reveals only two other unique structural motifs, a rather unusual [Cr_3_Ni_2_] linear complex [24], and an ‘S-shaped’ [Cr_12_Ni_3_] chain [25]. We have previously reported a metalloligand approach that enabled us to synthesise high-nuclearity heterometallic coordination capsules of paramagnetic transition metal ions in a modular and predictable fashion [26,27,28,29]. This metalloligand, based on the tritopic [M^III^L_3_] moiety shown in Figure 1 (HL = 1-(4-pyridyl)butane-1,3-dione), features a tris(acac) coordinated octahedral transition metal ion, in which the ligand is functionalised with a *p*-pyridyl donor group. In the *fac*-isomer of this metalloligand, the three N-donor groups are orientated in such a way that combination with a square-planar metal ion leads to the entropically favoured self-assembly of a cubic structure [30]. Herein, we report the syntheses, structures, and magnetic properties of three novel tetradecanuclear [Cr^III^_8_Ni^II^_6_]^n+^ cubes, namely [Cr^III^_8_Ni^II^_6_L_24_(H_2_O)_12_](NO_3_)_12_ (**1**), [Cr^III^_8_Ni^II^_6_L_24_(MeCN)_7_(H_2_O)_5_](ClO_4_)_12_ (**2**), and [Cr^III^_8_Ni^II^_6_L_24_Cl_12_] (**3**), which join the growing family of [M^III^_8_M^II^_6_] cubes constructed from [M^III^L_3_] and a variety of M^II^ salts (M^III^ = Cr, Fe; M^II^ = Cu, Co, Mn, Pd) [26,27,28,29].

## 2. Materials and Methods

### 2.1. Synthesis

1-(4-pyridyl)butane-1,3-dione (HL) and the metalloligand [Cr^III^L_3_] were prepared by previously published procedures [26,31,32]. All reactions were carried out under aerobic conditions. Solvents and reagents were used as received from commercial suppliers. Caution: perchlorate salts of metal complexes with organic ligands are potentially explosive.

Synthesis of [Cr^III^_8_Ni^II^_6_L_24_(H_2_O)_12_](NO_3_)_12_ (**1**). To a solution of [Cr^III^L_3_] (54 mg, 0.1 mmol) in 10 mL of dichloromethane, a solution of Ni(NO_3_)_2_∙6H_2_O (30 mg, 0.1 mmol) was added in 10 mL of methanol. The solution was stirred for 18 h before being filtered and allowed to stand. Dark orange X-ray quality crystals were obtained from the diffusion of diethyl ether into the mother liquor. Yield of (**1**) = 69%. Elemental analysis (%) calculated (found): C 46.16 (46.01) H 3.87 (3.78) N 8.97 (8.63).

Synthesis of [Cr^III^_8_Ni^II^_6_L_24_(MeCN)_7_(H_2_O)_5_](ClO_4_)_12_ (**2**). To a solution of [Cr^III^L_3_] (108 mg, 0.2 mmol) in 10 mL of dichloromethane, a solution of Ni(ClO_4_)_2_∙6H_2_O (73 mg, 0.2 mmol) was added in 10 mL of acetonitrile. The solution was stirred for 18 h before being filtered and allowed to stand. Brown X-ray quality crystals were obtained after 5 days from the diffusion of pentane into the mother liquor. Yield of (**2**) = 81%. Elemental analysis (%) calculated (found): C 44.34 (44.06) H 3.61 (3.59) N 6.97 (7.11).

Synthesis of [Cr^III^_8_Ni^II^_6_L_24_Cl_12_] (**3**). To a solution of [Cr^III^L_3_] (108 mg, 0.2 mmol) in 10 mL of dichloromethane, a solution of NiCl_2_ (20 mg, 0.15 mmol) was added in 10 mL of tetrahydrofuran. The solution was stirred for 18 h before being filtered and allowed to stand. Brown X-ray quality crystals were obtained after room temperature evaporation of the mother liquor for 5 days. Yield of (**3**) = 58%. Elemental analysis (%) calculated (found): C 51.01 (50.79) H 3.81 (3.71) N 6.61 (6.68).

### 2.2. Crystallographic Details

Single-crystal X-ray diffraction data were collected for (**1**)–(**3**) at *T* = 100 K on a Rigaku AFC12 goniometer equipped with an enhanced sensitivity (HG) Saturn 724+ detector mounted at the window of an FR-E+ Superbright MoKα rotating anode generator with HF Varimax optics (70 μm focus) [33]. The CrysalisPro software package was used for instrument control, unit cell determination, and data reduction [34]. Due to very weak scattering power, single-crystal X-ray diffraction data for (**1**) and (**2**) were collected at *T* = 30.15 K using a synchrotron source (λ = 0.6889 Å) on the I19 beam line at Diamond Light Source on an undulator insertion device with a combination of double crystal monochromator, vertical and horizontal focussing mirrors, and a series of beam slits. The same software as above was used for data refinement. Crystals of all samples were sensitive to solvent loss, which resulted in crystal delamination and poor-quality X-ray diffraction data. To slow down crystal degradation, crystals of (**1**)–(**3**) were “cold-mounted” on MiTeGen Micromounts^TM^ at *T* = 203 K using Sigma-Aldrich Fomblin Y^®^ LVAC (3300 mol. wt.), with the X-Temp 2 crystal cooling system attached to the microscope [35]. This procedure protected crystal quality and permitted collection of usable X-ray data. Unit cell parameters in all cases were refined against all data. Crystal structures were solved using Intristic Phasing as implemented in SHELXT [36]. All non-hydrogen atoms were refined with anisotropic displacement parameters, and all hydrogen atoms were added at calculated positions and refined using a riding model with isotropic displacement parameters based on the equivalent isotropic displacement parameter (U_eq_) of the parent atom. All three crystal structures contain large accessible voids and channels that are filled with diffuse electron density belonging to uncoordinated solvent, whose electron contribution was accounted for by the PLATON/SQUEEZE routine ((**1**) and (**2**)) [37], or by the SMTBX solvent masking routine, as implemented in OLEX2 software (**3**). To maintain reasonable molecular geometry, DFIX restraints were used in all three complexes.

Crystal Data for [Cr^III^_8_Ni^II^_6_L_24_(H_2_O)_12_](NO_3_)_12_ (**1**). C_216_H_216_Cr_8_N_24_Ni_6_O_60_, M_r_ = 4876.38, monoclinic, a = 25.754(3) Å, b = 41.336(5) Å, c = 43.217(5) Å, α = 90°, β = 90.6450(10)°, γ = 90°, V = 46,004(9) Å^3^, Z = 4, *P*2_1_/*n*, D_c_ = 0.704 g cm^−3^, µ = 9.18 mm^−1^, T = 100.15(10) K, 370,995 reflections measured, 81,102 unique (R_int_ = 0.1902), which were used in all calculations, wR_2_ (all data) = 0.3687, and R_1_ [I > 2(I)]= 0.1242. CCDC 1977309.

Crystal Data for [Cr^III^_8_Ni^II^_6_L_24_(MeCN)_7_(H_2_O)_5_](ClO_4_)_12_ (**2**). C_230_H_218_Cr_8_N_31_Ni_6_O_54_, M_r_ = 5048.60, monoclinic, a = 25.788(6) Å, b = 41.606(9) Å, c = 45.869(11) Å, α = 90°, β = 90.785(2)°, γ = 90°, V = 49,210(20) Å^3^, Z = 4, *P*2_1_/*n*, D_c_ = 0.681 g cm^−3^, μ = 0.412 mm^−1^, T = 100.15(10) K, 391,278 reflections measured, 85,150 unique (R_int_ = 0.2371), which were used in all calculations, wR_2_ (all data) = 0.4444, and R_1_ [I > 2(I)] = 0.1521. CCDC 1977311.

Crystal Data for [Cr^III^_8_Ni^II^_6_L_24_Cl_12_] (**3**). C_216_H_192_Cl_12_Cr_8_N_24_Ni_6_O_48_, M_r_ = 5085.58, triclinic, a = 28.171(16) Å, b = 30.225(16) Å, c = 32.40(2) Å, α = 72.27(6)°, β = 72.08(6)°, γ = 64.04(6)°, V = 22,417(27) Å^3^, Z = 2, *P*-1, D_c_ = 0.753 g cm^−3^, μ = 0.543 mm^−1^, T = 100.0(1) K, 134,344 reflections measured, 66,105 unique (R_int_ = 0.1446), which were used in all calculations, wR_2_ (all data) = 0.5544, and R_1_ [I > 2(I)] = 0.1938. CCDC 1977312.

### 2.3. Magnetic and Spectroscopic Measurements

Direct current (dc) susceptibility and magnetisation data were measured on powdered, polycrystalline samples of (**1**) using a Quantum Design SQUID MPMS-XL magnetometer, operating between 1.8 and 300 K for dc applied magnetic fields ranging from 0 to 5 T. X-band EPR spectra were collected on powdered microcrystalline samples of (**1**) using a Bruker EMX spectrometer at the EPSRC UK National EPR Facility at The University of Manchester.

## 3. Results and Discussion

### 3.1. Structural Description

**Figure 2:**
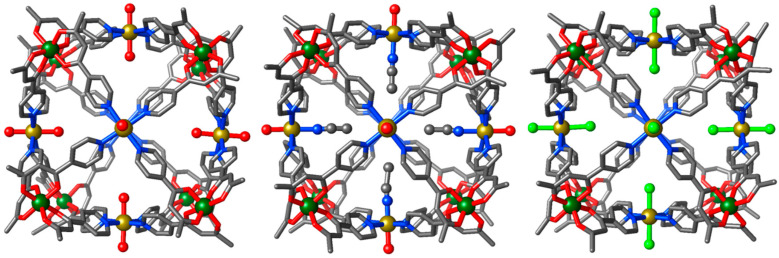
From left to right, molecular structures of (**1**), (**2**), and (**3**). Colour code: CrIII = green, NiII = yellow, O = red, N = blue, Cl = light green, C = grey. H-atoms have been omitted for clarity.

The heterometallic cubes [Cr^III^_8_Ni^II^_6_L_24_(H_2_O)_12_](NO_3_)_12_ (**1**), [Cr^III^_8_Ni^II^_6_L_24_(MeCN)_7_(H_2_O)_5_](ClO_4_)_12_ (**2**), and [Cr^III^_8_Ni^II^_6_L_24_Cl_12_] (**3**) were formed from the reaction of [Cr^III^L_3_] with the corresponding Ni^II^ salt in CH_2_Cl_2_/MeOH, CH_2_Cl_2_/MeCN, and CH_2_Cl_2_/THF, respectively. All three structures (Figure 2) reveal a similar [Cr^III^_8_Ni^II^_6_] cube-like metallic skeleton, with the eight Cr^III^ ions located at the corners and the six Ni^II^ ions located on the faces, approximately 1.4–2.3 Å above the Cr⋯Cr⋯Cr⋯Cr plane. The internal cavity volume of the cube is approximately 1400 Å^3^.

The Cr^III^ ions are all octahedral, possessing {CrO_6_} coordination spheres with Cr^III^-O distances between 1.9 and 2.2 Å, and *cis*/*trans* angles in the range 82.7–97˚/171.3–179.4˚, respectively. The equatorial positions of the octahedral Ni^II^ ions are occupied by four pyridyl donors from four distinct [Cr^III^L_3_] subunits, with Ni^II^–N distances in the range 1.9–3.0 Å. For (**1**) and (**2**), the axial positions are occupied by twelve water and twelve acetonitrile/water molecules (Ni^II^-O ≈2.1 Å; Ni^II^-N ≈2.1 Å), respectively. The cubes are therefore cationic (12+). The charge balancing nitrate or perchlorate anions for (**1**) and (**2**) respectively, are located both within the central cavity of the cube and in the void spaces between cubes. In contrast to (**1**) and (**2**), complex (**3**) is neutral, with the axial positions of the Ni^II^ ions occupied by chloride anions (Ni^II^-Cl ≈2.7 Å).

**Figure 3:**
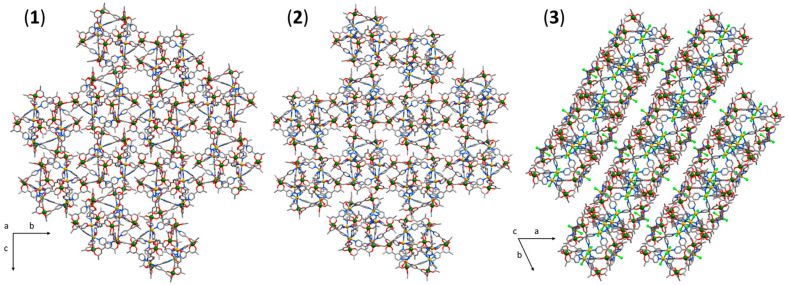
Packing diagrams of (**1**)–(**3**) viewed down the *a*-, *a*-, and *c*-axis, respectively. Colour code as in Figure 1.

There are several close intermolecular contacts (Figure 3) between the cages in the extended structures of (**1**)–(**3**). In (**1**), the closest inter-cluster contact is between the aromatic protons of the pyridyl group and the O-atom (2.3 Å) of a neighbouring L^−^ ligand. In (**2**) and (**3**), the closest contact is between the protons of the metalloligand methyl group, and the O-atom of a neighbouring L^−^ ligand (2.3 Å) and the protons of a neighbouring methyl group (2.3 Å), respectively. Several other close inter-cluster contacts between neighbouring cubes exist, for example: Ar-H···O ≈2.5 Å and C-H···O ≈2.7 Å for (**1**), H_2_C-H···O ≈2.5 Å and H_2_O···H-CH_2_ ≈2.7 Å for (**2**), and Ar-H···Cl ≈2.7 Å and C-H···Cl ≈2.8 Å for (**3**).

### 3.2. Magnetic Properties

**Figure 4:**
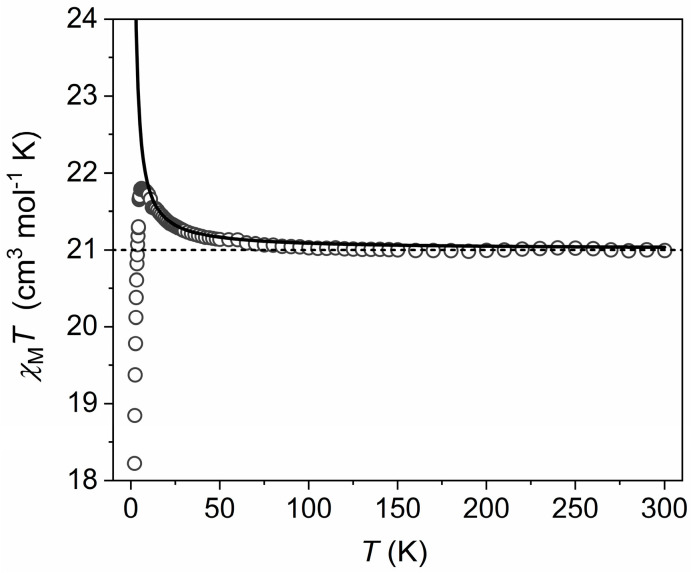
Plot of *χ*_M_*T* (open circles) versus *T* for complex (**1**), with the sum of the Curie constants of the uncorrelated ions and the best-fit data represented by the dashed and solid lines, respectively.

As complexes (**1**)–(**3**) are structurally analogous, and for the sake of brevity, we discuss only the behaviour of a representative example, complex (**1**). The dc molar magnetic susceptibility, *χ*_M_, of a polycrystalline sample of (**1**) was measured in an applied magnetic field, *B*, of 0.1 T, over the 2–300 K temperature, *T*, range. The experimental results are shown in Figure 4 in the form of the *χ*_M_*T* product versus temperature, where *χ*_M_ = *M*/*B*, and *M* is the magnetization of the sample. Due to the loss of lattice solvent during the evacuation of the sample chamber of the SQUID magnetometer, leading to an uncertainty in the molar mass of the measured sample, the *T* = 300 K *χ*_M_*T* product of (**1**) was scaled to 21.00 cm^3^ mol^−1^ K, the expected value from the sum of Curie constants for a [Cr^III^_8_Ni^II^_6_] unit, with *g*_Cr_ = *g*_Ni_ = 2.0, where *g*_Cr_ and *g*_Ni_ are the *g*-factors of Cr^III^ and Ni^II^, respectively. Note that this rescaled value has a maximum deviation of 15% from the unscaled data.

**Figure 5:**
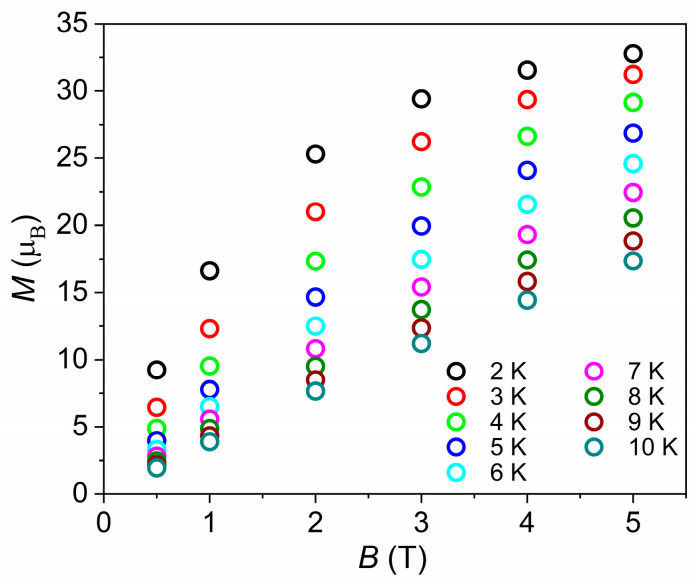
VTVB magnetisation data for (**1**) in the temperature and field ranges *T* = 2–10 K and *B* = 0.5–5 T.

Upon cooling, the value of *χ*_M_*T* remains essentially constant to approximately *T* = 75 K, where it begins to increase, reaching a maximum of 21.8 cm^3^ mol^−1^ K at *T* = 6 K. Below this temperature, *χ*_M_*T* falls rapidly to a minimum value of 18.5 cm^3^ mol^−1^ K at *T* = 2.0 K. The behaviour is suggestive of weak ferromagnetic exchange between the Cr^III^ and Ni^II^ ions, with the decrease in *χ*_M_*T* below 6 K attributed to intermolecular antiferromagnetic exchange interactions, and/or zero-field splitting (zfs) effects primarily associated with the Ni^II^ ions. Quantitative analysis of the susceptibility data via standard matrix diagonalization techniques is non-trivial due to the large nuclearity of the cluster and the associated enormous dimensions of the spin-Hamiltonian matrices. Even the total spin (*S*) block matrices used in approaches based on Irreducible Tensor Operator algebra are of larger dimensions than what is realistic for exact numerical matrix diagonalization. Previously, we reported the use of computational techniques, known in theoretical nuclear physics as statistical spectroscopy [38], to analyse the structurally similar [M^III^_8_M^II^_6_]^n+^ (M^III^ = Cr, Fe; M^II^ = Co, Cu, Ni; n = 0–12) cubes [26,27,28]. We now extend this methodology to quantify the exchange interactions present in (**1**). Due to the fact that the influence of the zfs of the Ni^II^ ions will mainly affect the measured properties at low temperatures, the use of the isotropic spin-Hamiltonian (1) is sufficient to model the exchange interactions between Cr^III^ and Ni^II^ ions in the *T* = 300–6 K region:

(1) $${\hat{H}}_{iso}=-2J_{\mathrm{Cr}-\mathrm{Ni}}\underset{\mathrm{all}Cr-Ni\mathrm{pairs}}{\sum }{\hat{S}}_{\mathrm{Cr}}\cdot {\hat{S}}_{Ni}+\mu_{B}Bg\underset{i}{\sum }{\hat{S}}_{i}^{Z}$$

with *i* running over all constitutive metal centres, *g* is the isotropic *g*-factor, *Ŝ* is a spin-operator, *J*_Cr-M_ is the isotropic exchange parameter between Cr^III^ and M^II^ centres, and μ_B_ is the Bohr magneton. We assume common *g*-factors for both Cr^III^ and Ni^II^ (*g*_Cr_ = *g*_Ni_ = 2.0) since the 300 K *χ*_M_*T* product of (**1**) was scaled to the sum of its Curie constants, as explained above. We neglect any *J*_Cr-Cr_ and *J*_Ni-Ni_ terms as these centres are not connected as first neighbours. Using Hamiltonian (**1**), *J*_Cr-Ni_ was determined to be +0.045 cm^−1^. Variable-temperature and variable-field (VTVB) magnetization studies of (**1**) collected in the *T* = 2–10 K and *B* = 0.5–5 T temperature and field ranges (Figure 5) are consistent with this picture. *M* reaches a value of 32.8 µ_B_ at *B* = 5 T and *T* = 2 K, approaching the saturation value of 36 µ_B_, consistent with relatively small exchange-induced splittings that lead to the *m_S_* = 18 projection of the *S* = 18 total spin state, being the ground state at the highest measured magnetic field. The weak ferromagnetic exchange between the d^3^ Cr^III^ ions and the d^8^ Ni^II^ ions is as one would expect, mediated via the 1-(4-pyridyl)butane-1,3-dione ligand [26,27,28].

### 3.3. EPR Spectroscopy

**Figure 6:**
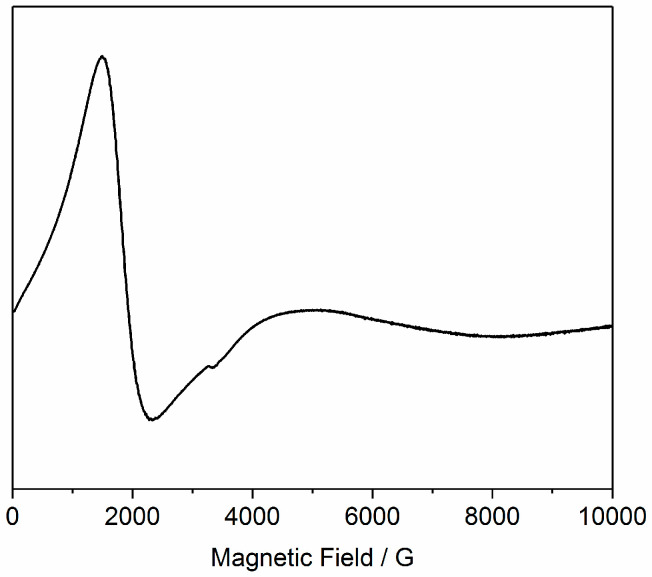
X-band (*ca*. 9.4 GHz) EPR spectrum of a powdered sample of (**1**) at 5 K.

X-band EPR spectra of a powdered sample of (**1**) at 5 and 10 K are dominated by a feature at ca. 2 kG (Figure 6). This is similar to spectra from the isolated [Cr^III^L_3_] complex, and related [Cr^III^_8_M^II^_6_] and [Cr^III^_2_M^II^_3_] species [26,29], and arises from the Cr^III^ (*S* = 3/2) ions with a near-axial zero-field splitting of |*D*_Cr_| ca. 0.5–0.6 cm^−1^. This is only consistent with a weak exchange interaction |*J*_Cr-Ni_| with respect to |*D*_Cr_|, and hence consistent with the magnetic data. There are no clear features arising from the Ni^II^ (*S* = 1) ions, which implies that |*D*_Ni_| must be much larger than the microwave energy. We also observed this for a related [Fe^III^_8_M^II^_6_] cube, which only showed EPR features due to Fe^III^ [28]. This is consistent with |*D*_Ni_| values of 5–10 cm^−1^ determined from magnetization studies of isolated [Ni^II^(pyridine)_4_X_2_] complexes [39], and with high-field EPR studies of Ni^II^ complexes with mixed N,O-donor sets [40].

## 4. Conclusions

We have shown that the modular self-assembly of [M^III^L_3_] metalloligands with simple M^II^ salts can be exploited to construct large heterometallic coordination compounds of Cr^III^ and Ni^II^. Compounds (**1**)–(**3**) join a growing family of [M^III^_8_M^II^_6_] cubes, where M^III^ = Cr and Fe and M^II^ = Cu, Co, Mn, Pd, and Ni. The ability to build families of isostructural complexes containing different combinations of paramagnetic (and diamagnetic) metal centres aids the qualitative and quantitative understanding of magnetic properties and the underlying structural parameters that govern behaviour. Examples of large, heterometallic cages in which the 3*d* metal ions can be exchanged with other 3*d* metal ions are extremely rare.

Magnetic susceptibility and magnetization data show the presence of weak ferromagnetic exchange between the Cr^III^ and Ni^II^ ions, with *J*_Cr-Ni_ = +0.045 cm^−1^. EPR spectroscopy is consistent with the exchange interactions being much weaker than the zero-field splittings of both the Cr^III^ and Ni^II^ ions.

## Data Availability

The data presented in this study are available on request from the corresponding authors.
